# Development, implementation, and evaluation of neonatal thermoregulation decision support web application

**DOI:** 10.1186/s12911-023-02302-4

**Published:** 2023-10-18

**Authors:** Raziyeh Beykmirza, Elahe Rastkar Mehrabani, Maryam Hashemi, Maryam Mahdizade Shahri, Reza Negarandeh, Maryam Varzeshnejad

**Affiliations:** 1 Nursing and Midwifery Care Research Center, School of Nursing and Midwifery, Tehran University of Medical Sciences, Tehran, Iran; 2 Clinical Research Development Center, Mahdiyeh Educational Hospital, Shahid Beheshti University of Medical Science, Tehran, Iran; 3 Nursing Faculty, Jahrom University Medical Sciences, Jahrom, Iran; 4 Department of Pediatric Nursing, School of Nursing and Midwifery, Shahid Beheshti University of Medical Sciences, Tehran, Iran

**Keywords:** Thermoregulation, Neonates, Decision support system, Electronic temperature recording

## Abstract

**Objectives:**

Thermoregulation is important for all age groups, and in neonates, it is considered a crucial event to adapt to extrauterine life. Therefore, using systems that provide frequent reminders in different ways in the field of thermoregulation can help thermal stability in neonates. The present study aimed to develop, implement, and evaluate a neonatal thermoregulation decision support system (DSS) as a web application.

**Methods:**

The present research was a multi-method study because it included the three phases of development, implementation, and evaluation of the neonatal thermoregulation decision support web application. In the system designing phase, the waterfall model is used. The second and third phases of the study, implementation, and evaluation, were conducted as a quasi-experimental study.

**Results:**

The results of this study were presented in two parts: the developed web application, and the results of the evaluation of the web application. The results of the statistical tests revealed that the use of the web application had a positive and significant effect on both the adjustment of the temperature of the incubator (maintaining the neutral temperature) and the maintenance of the temperature of the neonate’s body (p = 0.000).

**Conclusions:**

These results indicate that a nurse’s sensitization and guidance with a neonatal thermoregulation decision support system can help to effectively neonate thermoregulation and the nurse has brought the temperature care close to the standard care based on the conditions of each neonate.

**Supplementary Information:**

The online version contains supplementary material available at 10.1186/s12911-023-02302-4.

## Introduction

Thermoregulation is important for all age groups, and in neonates, it is considered an important event to adapt to extrauterine life [1]. It is one of the most important biological adjustments of the body [2]. Regulation of the thermal environment of neonates has long been considered one of the vital aspects of care [3]. The reasons for the increased sensitivity to thermoregulation problems in the neonate include a higher proportion of body surface to weight, more evaporation due to the immaturity of the skin, lack of subcutaneous fat tissue that acts as a temperature insulator, muscular weakness, and insufficiency of the regulation of dermal blood circulation [1]. Both hypothermia and hyperthermia exert many short-term and long-term negative effects on neonates [4] and can cause many problems, including weakness and lethargy, failure to weight gain, apnea, and even death of the neonate [5]. Despite the great advances in knowledge and equipment and supportive methods of thermoregulation, the issue of thermoregulation in neonates, especially in premature neonates, is still very challenging. In high-risk environments such as neonatal intensive care units, maintaining a neutral thermal environment is a serious challenge that can affect mortality and complications in neonates at all gestational ages [6]. The purpose of neonatal care in an incubator is to maintain a neutral temperature. A neutral temperature is a temperature that does not increase oxygen consumption and the metabolic needs of the neonate’s body [5]. Thermoregulation is a complex physiological function that is influenced by many factors. Controlling the environmental temperature is one of the main factors in regulating the body temperature of neonates [7]. Therefore, the purpose of caring for a neonate in an incubator is to help maintain a neutral temperature by controlling the environmental temperature [6].

Neonatal nurses should have the ability to recognize neonates at risk for thermal instability to prevent, quick and early diagnosis, and management of hypothermia and hyperthermia [5]; in addition, accurate monitoring of the body temperature of neonates is very important due to its relationship with the mortality and morbidity of neonates [8]. Research has shown that providing continuous training to neonate nurses and using systems that provide frequent reminders in different ways in the field of thermoregulation can help thermal stability in neonates [9]. Also using guidelines is one of the useful ways to maintain the neutral temperature of every neonate [6]. Yet, the complexity of the guidelines for neonatal thermoregulation is one of the most important challenges for nurses, because to maintain a neutral temperature, the environmental temperature of each neonate should be determined based on the daily neonatal weight and age (based on an hour or day), and then adjusted regularly based on changes in daily weight and age. In so doing, nurses must either regularly refer to the temperature adjustment guideline and adjust the temperature of their neonate’s incubator based on the guide table, which is very time-consuming, or they must memorize the adjustment temperatures for each birth weight and age, which is practically impossible.

Nowadays to help in clinical decision-making based on guidelines and reduce patient safety challenges, the use of clinical decision support systems has been considered. Clinical decision support systems are a type of electronic system in clinical care [10] that are designed to support the care team members in the process of reviewing and updating new clinical evidence [11]. The need for clinical decision support systems is undeniable because most activities in the field of health care require decision-making processes [12]. In recent years, clinical decision-support systems have been increasingly considered to ensure patient safety and support all stages of clinical decision-making [13]. The studies conducted on the effectiveness of the clinical decision support systems report results such as improving process outputs like increasing the acceptance of relevant rules and principles, improving teamwork and communication, as well as saving money [14]. Moreover, the use of clinical decision support systems can improve the knowledge of health caregivers [15] and affect the quality of nursing documentation and patient safety [16]. Hence, to improve the quality of patient care and compliance with regulatory standards, it is recommended to use clinical decision support systems [17].

The noteworthy point in this context is that, despite the development of many clinical decision support systems in the field of nursing care improvement, no system has yet been designed that can help nurses in the field of neonatal thermoregulation; consequently, the present study was conducted to design, implement and evaluating the electronic decision support system for neonatal thermoregulation.

## Methods

**Fig. 1:**
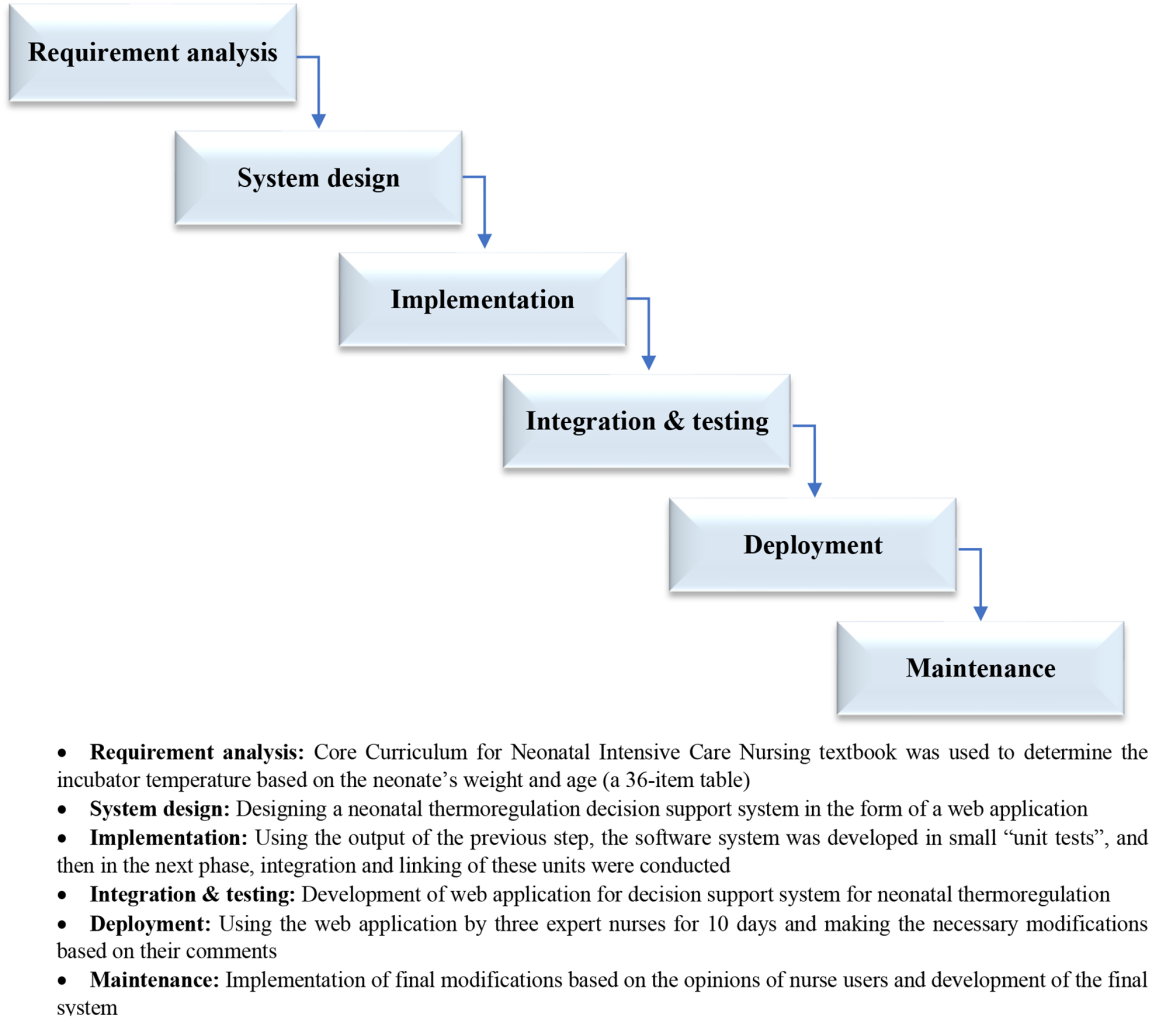
Thermoregulation decision support system web application development steps based on the waterfall model

The present research was a multi-method study because it included the three phases of designing, implementing, and evaluating the decision support system with each phase having its own methodology. In the system development phase, the waterfall model is used, which is one of the most famous methods of development of information technology products. The waterfall model was chosen in this study because of its special advantages: 1- It has specific steps, 2- The stages of the project can proceed in parallel, 3- From the beginning of the development of the system, the end of the work is clear, 4- It is easy to evaluate the project step by step. The waterfall model includes six interrelated stages, including 1- Requirement analysis, 2- System design, 3- Integration and testing, 4- Implementation, 5- Establishment or deployment, and 6- Maintenance [18] (Fig. 1). The second and third phases of the study, implementation, and evaluation, were conducted as a quasi-experimental study.

### Development stage

In this study, after approving the proposal and obtaining the code of ethics no.: IR.TUMS.MEDICINE.REC.1400.1525, the guideline about neonatal thermoregulation was derived from the latest edition of the CORE CURRICULUM FOR NEONATAL INTENSIVE CARE NURSING textbook [5], which is the main reference for neonatal nursing care. In this book, a detailed table is provided for setting the temperature of the incubator for neonates based on the neonate’s weight and age (Supplementary file# 1).

Considering that the neonatal thermoregulation guideline is a 36-item table that it is not possible to memorize the nurse who wants to adjust the neonate’s temperature, and this is one of the main reasons for the inappropriate temperature management of neonates Requirement analysis: First step of waterfall model). In the next step, System design: The second step of the waterfall model؛ the content supposed to be converted into a web application was drawn on paper with full details in the form of a program (such as program pages, information on each page, how to link pages, program output, etc.); then, the paper program was converted into a web application with the cooperation of an expert computer engineer. In the next step, Implementation: the third step of the waterfall model, each of the different parts of the web application was used individually by three expert nurses for a week, and based on their opinions, fixed some little problems and entered the fourth stage of web application development: Integration and testing. In this step, the whole web application was used as a pilot study by three volunteer nurses again for a week, and using the opinions of nurses, the web application was modified and the final version of the web application was developed. In the last stage of the system development, the web application was delivered to all the nurses of the neonatal intensive care unit in a maternal and neonatal hospital, and the researchers assumed the responsibility of supporting the web application. The output of this research is a clinical decision support system as a thermoregulation web application that not only allows the nurse to set the appropriate temperature based on the specific conditions of each neonate by providing standard guidelines but also, records how to regulate the temperature of neonates, and provides the possibility of evaluating the nurse’s performance.

### Implementation stage

The present research was conducted in Mahdieh Hospital in Tehran, which is a referral center for maternal and neonatal care and has two neonatal intensive care units. The researchers explained the objectives of the project to the nursing colleagues and identified the eight nurses who volunteered to cooperate, in the study. The 100 neonates were calculated as the sample size that was determined with the G Power software, considering a test power of 90% and using the study by Wang (2018) [19]. The validity of this software has been emphasized in numerous studies.

**Fig. 2:**
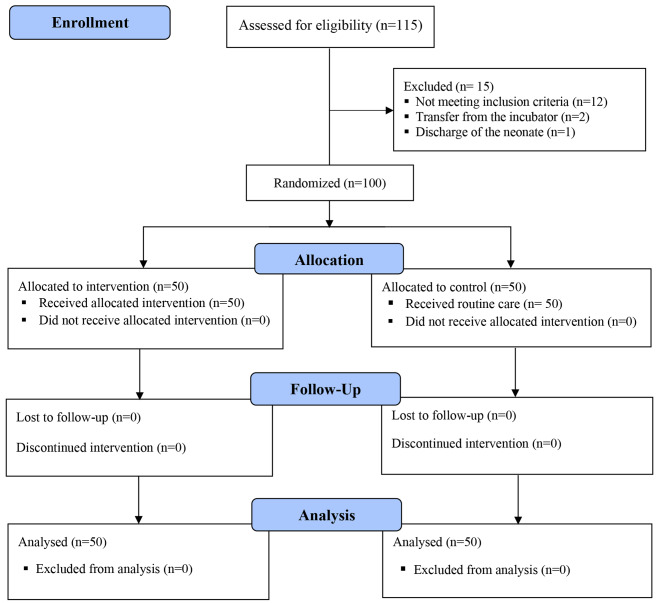
CONSORT flow diagram

After obtaining informed consent from the parents of the neonates with consideration of inclusion criteria, they were randomly divided into two control and intervention groups (Fig. 2).

The inclusion criteria for neonates in the study were: incubator care with the ability to adjust the temperature, the neonate’s not having any acute surgical condition, no phototherapy of the neonate (because the phototherapy device probably affects the thermoregulation of the neonate), no sepsis or infectious diseases, and the neonate not being in the end stages of life. The exclusion criteria were: discharge of the neonate or transferring the neonate out of the incubator before at least three temperature records for the neonate, the neonate suffering from sepsis or infectious disease during the implementation of the study. Neonates in the control group received thermoregulation care based on the hospital routine care, and the intervention group received thermoregulation routine care, and the daily neonatal temperature incubator adjustment was done based on the guidelines recommended by the web application. To minimize human error and achieve more detailed results, each neonate was assessed three times at an interval of at least 6 and most 24 h. Finally, 158 temperature records were done in the control group and 221 temperatures were recorded in the intervention group. All neonates of the control and intervention groups were cared for by the same eight volunteer nurses, and each nurse cared for at least 4 and at most 5 neonates from each of the control and intervention groups. Also, the parents were assured that if they were not satisfied, they could withdraw from participating in the study at any time or stage. 15 neonates were excluded during the study due to the exclusion criteria.

### Evaluation stage

The developed web application was evaluated from two aspects. First, by determining the effect of using the web application in maintaining the neutral temperature (incubator of the neonate), and maintaining the skin temperature of the neonate in the normal range, and second, by the satisfaction of the nurse users with the neonatal thermoregulation web application. It is worth noting that maintaining the neutral temperature of the neonate’s environment was one of the important points of attention in this research because this issue is usually overlooked. In fact, in many cases, the neonate maintains the body temperature in the normal range by consuming oxygen and increasing the metabolism; this shows the lack of attention to maintaining the neutral temperature of the neonate’s incubator. To evaluate the effect of the web application on the thermoregulation of neonates, a neonatal information registration checklist including gestational age, neonatal weight at the time of temperature recording, postnatal age in hours at the time of temperature recording, incubation temperature, and skin temperature of the neonate was prepared. Furthermore, the Mobile Application Rating Scale was used to evaluate the thermoregulation web application. This scale can be used to evaluate the general level of user satisfaction with all applications that are installed on mobile phones. This scale includes 5 parts and 23 items. Part A, entitled ‘Engagement’, assesses the accurate directing of users by asking 5 questions. Part B, entitled ‘Functionality’, assesses the performance of the program, easy learning, the logic of different parts of the program, and the general appearance of the software using 4 items. Part C, entitled ‘Aesthetics’, assesses the design and graphics of the program, the overall visual appeal of the program, the color scheme, and coordination and integration of the program using 3 items. Part D, entitled ‘Information’ assesses information and quality (including texts, feedback, measurements, and referrals) from reliable sources using 7 items. Part E, entitled ‘App subjective quality’ assesses issues like the possibility of recommending the program to others and buying the program, etc., using 4 items. The scoring of the scale is done using a 5-point Likert scale. Of course, each option is specified in the item. Finally, all data was entered into the SPSS version 16, and the chi-square test was used to compare the temperatures in the two control and intervention groups and to explain the nurses’ satisfaction with the neonatal thermoregulation web application the report of the overall Mean ± SD score and subscales scores of the MARS scale was used.

## Results

The results of this study were presented in two parts: the developed neonatal thermoregulation web application, and the results of the evaluation of the neonatal thermoregulation web application. In the developed web application, the neonatal nurse (user) entered the system with the user’s name and password defined for her in advance. Each nurse enters the patient’s information (date and time of birth) into the system once, and then every time the patient’s name is searched, the system automatically calculates and displays the neonate’s age. The demographic characteristics of the 8 nurses participating in the study are presented in Table 1.

**Table 1:** Demographic characteristics of participating nurses (n = 8)

Demographic characteristics	Category	n	%
Sex	Female	8	100
	Male	0	0
Age	22 to 27 y	4	50
	27 to 32 y	2	25
	32 to 37 y	2	25
Educational degree	Bachelor’s degree	7	87.5
	Master’s degree	1	12.5
Nursing experience	1 to 5 y	5	62.5
	5 to 10 y	3	37.5
Neonatal nursing experience	1 to 5 y	4	50
	1 to 5 y	4	50

**Fig. 3:**
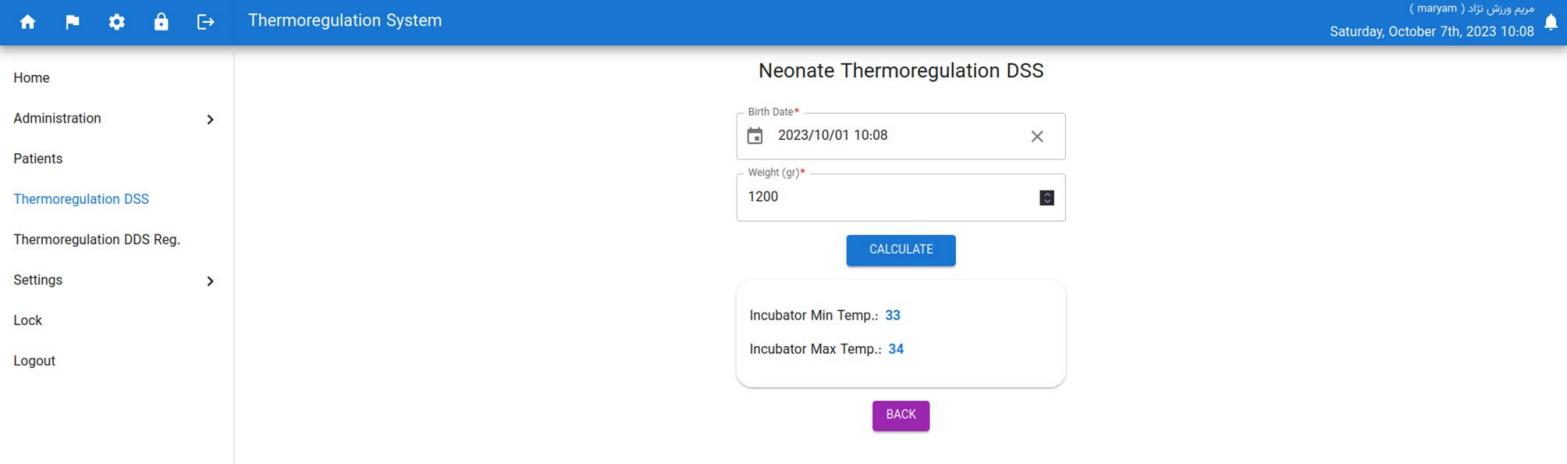
Calculation of the minimum and maximum temperature of the incubator based on the neonatal age and weight (Web application page)

Nonetheless, the daily weight of the neonate should be entered by the nurse due to the changing weight of the neonate every day. After choosing the neonate’s name and entering the daily weight, the system will show the nurse the minimum and maximum temperature that the incubator should be set to, according to the neonate’s age and weight (Fig. 3).

**Fig. 4:**
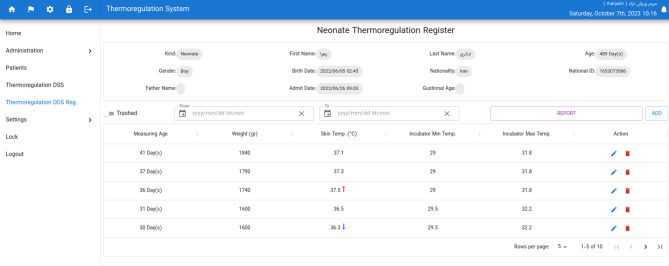
Temperature recording table with downward or arrows (Web application page)

The nurse sets the temperature of the incubator to the suggested temperature by the system takes the skin temperature of the neonate 15 to 20 min later and records it in the system. According to the brochure of the available incubators, it takes about 5 to 10 min to reach the air incubator temperature to set the temperature incubator and also about 5 to 10 min for the skin neonate’s temperature to adjust to the air temperature, and therefore the axillary skin temperature of neonates was recorded by a digital thermometer about 20 min after setting the air temperature of the incubator. At this time, the system displays the normal range of body temperature, the chart recorded based on the previously recorded temperatures, and the necessary warnings to the nurse. For example, one downward arrow next to the recorded body temperature indicates mild hypothermia, two downward arrows indicate moderate hypothermia, and similarly, three downward arrows next to the neonate’s body temperature value indicate severe hypothermia. Similarly, hyperthermia was immediately displayed to the nurse with an upward arrow based on the recorded value (Fig. 4).

**Fig. 5:**
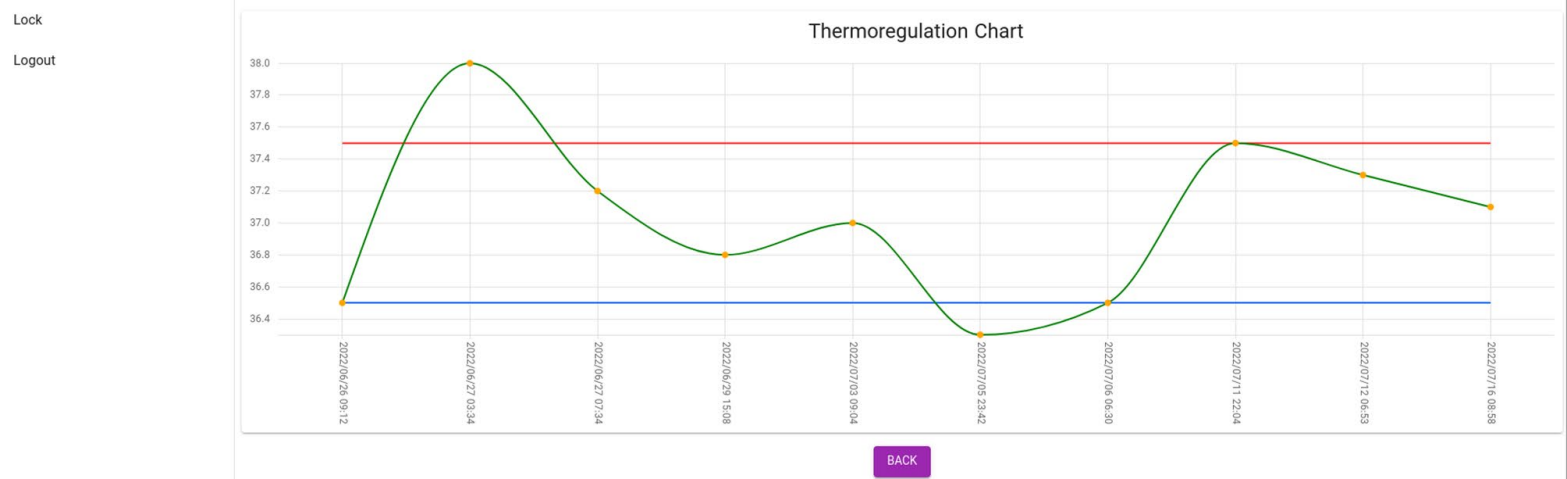
Neonate’s body temperature chart (Web application page)

**Fig. 6:**
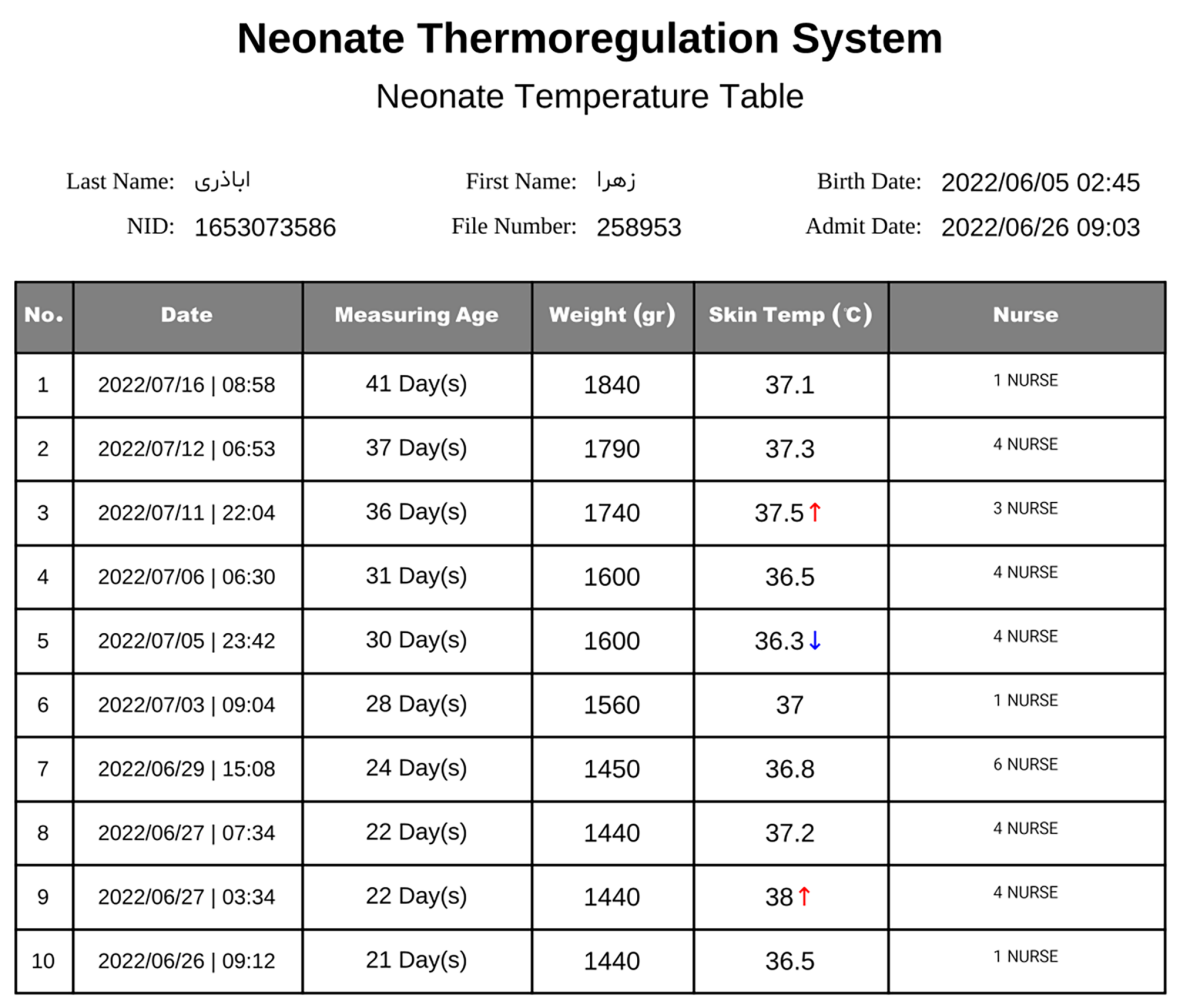
Report of the whole process of temperature management (Web application page)

Besides, the system draws the neonate’s body temperature chart automatically and indicates the cases where the chart is higher or lower than the normal range by color (Fig. 5). Finally, the report of the whole process of temperature management of the neonate is displayed in the form of a PDF page with the ability to print (Fig. 6).

The second part of the results was the statistical outputs, which were divided into three parts: 1- The effect of using the web application on maintaining the neutral temperature of the environment (incubator), 2- The effect of using the application on maintaining the temperature of the neonate’s body, 3- The result based on the satisfaction of the nurse users. The results of the statistical tests revealed that the use of the web application had a positive and significant effect on both the adjustment of the temperature of the incubator (maintaining the neutral temperature) and the maintenance of the temperature of the neonate’s body. In this way, no cases of hypothermia were recorded in the intervention group, and the number of cases of hyperthermia in the intervention group was less than that of the control group. Also, the use of the web application was 100% successful in maintaining the neutral temperature of the environment (Table 2).

**Table 2:** Frequency distribution and percentage of body temperature and incubator (neutral temperature) in two intervention and control groups

Variable	Temperature classification	Control group N(%)	Intervention group N(%)	P Value
Infant temperature	Hyperthermia	40(25.3)	14(6.3)	0.000**
	Normal	107(67.8)	207(93.7)	
	Hypothermia	11(6.9)	0(0)	
Incubator temperature (Natural temperature)	Hyperthermia	23(14.5)	0(0)	0.000**
	Normal	106(67.2)	221(100)	
	Hypothermia	29(18.3)	0(0)	

^**^ **Chi-squared test**

It is worth noting that an axillary temperature less than 35.5 is defined as neonatal hypothermia and more than 37.5 degrees as neonatal hyperthermia [20]. Moreover, considering that the guidelines for adjusting the body temperature of neonates are based on the two criteria of weight and age after birth at the time of temperature adjustment, these two variables were recorded in both groups; in this regard, the control and intervention groups were not statistically significantly different (Table 3).

**Table 3:** Frequency and percentage of weight and age after the birth of neonates in two control and intervention groups

Variable	Classification	Intervention group N(%)	Control group N(%)	P value
**Neonatal age**	0–96 h	35 (15.8)	26(16.5)	0.200^***^
	4–14 days	71 (32.1)	74(46.8)	
	2–6 weeks	115 (52.1)	58(36.7)	
**Neonatal weight**	Less than 1200	69 (31.2)	43(27.2)	0.257 ^***^
	1200 to 1500	7 (3.2)	10(6.3)	
	1501 to 2500	108 (48.9)	78(49.4)	
	More than 2500	37 (16.7)	27(17.1)	

***Mann-Whitney U Test

The result was based on the satisfaction of the nurse users with the “Mobile Application Rating Scale (MARS)” Among the dimensions of the scale, the” Information” dimension had the highest mean score (29.12 ± 5.38) and the “Aesthetics” dimension had the lowest score mean score (12.25 ± 2.12). Among the subjects of the MARS scale " Target group”, “Ease of use” and “Quantity of information “, had the highest score of 36 and the lowest score was about the “Would you pay for this app?” question with a score of 18 (Table 4).

**Table 4:** Satisfaction of the participating nurses with the temperature adjustment decision support system

Dimensions	Items	Scores N(%)					Total score of each item	Final scores for each dimension		
		**1**	**2**	**3**	**4**	**5**		**Mean ± SD**	**Min**	**Max**
Engagement	Entertainment	0 (0)	0(0)	5(62/5)	1(12.5)	2(25)	29	19.63 ± 4.30	14	25
	Interest	0 (0)	1(12.5)	2(25)	2(25)	3(37.5)	31			
	Customization	0 (0)	1(12.5)	2(25)	2(25)	3(37.5)	31			
	Interactivity	0 (0)	0 (0)	5(62.5)	0(0)	3(37.5)	30			
	Target group	0 (0)	0(0)	1(12.5)	2(25)	5(62.5)	36			
Functionality	Performance	0 (0)	0(0)	1(12.5)	5(62/5)	2(25)	33	17.25 ± 3.10	12	20
	Ease of use	0 (0)	0(0)	2(25)	0(0)	6(75)	36			
	Navigation	0 (0)	0(0)	2(25)	2(25)	4(50)	34			
	Gestural design	0 (0)	0(0)	2(25)	1(12.5)	5(62.5)	35			
Aesthetics	Layout	0 (0)	0(0)	0(0%)	5(62.5)	3(37.5)	35	12.25 ± 2.12	10	15
	Graphics	0 (0)	0(0)	3(37.5)	2(25)	3(37.5)	32			
	Visual appeal	0 (0)	0(0)	3(37.5)	3(37.5)	2(25)	31			
Information	Accuracy of app description	0 (0)	0(0(	1(12.5)	4 (50)	3(37.5)	34	29.12 ± 5.38	21	35
	Goals	0 (0)	0(0(	3(37.5)	1(12.5)	4(50)	33			
	Quality of information	0 (0)	0(0(	4(50)	2(25)	2(25)	30			
	Quantity of information	0 (0)	0(0)	0(0)	4 (50)	4(50)	36			
	Visual information	0 (0)	1(12.5)	3(37.5)	1(12.5)	3(37.5)	30			
	Credibility	0 (0)	0(0	2(25)	1(12.5)	5(62.5)	25			
	Evidence base	0 (0)	0(0)	2(25)	1(12.5)	5(62.5)	25			
App subjective quality	Would you recommend this app to people who might benefit from it?	0 (0)	0(0)	2(25)	1(12/5)	5(62/5)	25	14.5 ± 2.97	10	17
	How many times do you think you would use this app in the next 12 months if it was relevant to you?	0 (0)	0(0)	3(37.5)	5(62.5)	0(0)	29			
	What is your overall star rating of the app?	0 (0)	0(0)	2(25)	2(25)	4(50)	34			
	Would you pay for this app?	**1**		**3**		**5**	18			
		3(37.5)		0(0)		5(62.5)				
Total	**Mean ± SD**	**Max**					**Min**			
	92.75 ± 14.82	112					76			

Also, to investigate the results of nurses’ satisfaction with the electronic decision support system, nurse colleagues were asked to record at least three temperature records for each neonate; yet, the results showed that in the intervention group, the number of recorded cases by the nurse was more than the predicted minimum. This suggests the attractiveness and user-friendliness of the temperature adjustment decision support system, which made the nurses check and register more than the required number for each neonate. For this reason, the number of registered cases in the intervention group was reported to be more than the number in the control group and the predicted sample size.

## Discussion

In analyzing the results of this study, several points should be mentioned. First, in most of the studies conducted in the field of neonatal thermoregulation, attention has been paid to the prevention and treatment of hypothermia, but less attention has been paid to neonatal hyperthermia [21–23]. Second, more importantly, despite the existence of guidelines and frequent recommendations of references and texts, in the reviewed studies, attention to maintaining the neutral temperature of the environment for the neonate has been a missing point and in most of the conducted research, if the neonate’s body temperature is reported in the normal range, the situation is considered desirable. Thus, it has been ignored that the neonate may have maintained the body temperature in the normal range by consuming energy and oxygen due to the lack of adjustment of the neutral temperature of the environment. This problem will lead to long-term complications such as insufficient neonatal weight gain, increased length of hospital stays due to insufficient weight gain, and other similar issues [5]. Third, the temperature of the incubator is not the only factor affecting the maintenance of the body temperature of the neonate and the neutral temperature in neonates admitted to the neonatal intensive care unit; rather, other factors such as the type of incubator, the humidity of the environment, whether the incubator is close or far from heating or cooling systems, the number of times it is opened and closed, the medical and clinical status of the neonate, and the amount of skin-to-skin contact between the neonate and the mother have also been effective in this regard [5]. The fourth point is that the use of thermoregulation decision-making systems has not been considered in any of the previous studies. The results of the present study demonstrated that the use of the web application for adjusting the neonate’s temperature was effective both in maintaining the neonate’s body temperature and in maintaining the neutral temperature. This point is important because in the present study, there is not any intervention such as Kangaroo mother care or covering the neonate, etc., and only by using the application, the nurse was able to adjust the temperature of the neonate’s incubator based on the guidelines. Besides, when recording the neonate’s body temperature, abnormal temperatures were reminded to the nurses immediately and other steps of temperature adjustment were left to the clinical judgment of the nurse. These results indicate that only a nurse’s sensitization and guidance can help to effectively thermoregulate the neonate and the nurse has brought the temperature care close to the standard care based on the conditions of each neonate and the available facilities and conditions. Additionally, the results of the present study showed that the use of the web application led to the fact that the body temperature of the neonates in the intervention group was significantly better than the control group. Also, in terms of setting the temperature of the incubator (neutral temperature), in the control group, despite setting the temperature based on the initial temperature of the skin, only 67% of the incubator temperature was in the correct range based on gestational age and daily weight; but this rate was 100% in the intervention group.

It is worth noting that the incidence and prevalence of neonatal hyperthermia and hypothermia have been reported very differently in the reviewed studies. For example, in the study by Yangthara et al., which was a longitudinal study performed during 2011–2015, the rate of hypothermia was 5.3 and hyperthermia was 21.4 at the time of admission of high-risk neonates [24]. In this regard, it should be pointed out that usually at the beginning of admission, neonates are not yet in a stable temperature condition. In addition, in a study conducted during 2016–2019 in the form of a longitudinal study for premature (under 37 gestational weeks) and low weight (under 2.5 kg) to determine the neonatal temperature at the time of transfer, the results showed that only 60% of the neonatal body temperature were in the normal range [25]. This amount was higher in both the control and intervention groups in the present study than in the study by Glen et al. Of course, in the present study, the neonates were all in the neonatal intensive care unit and were in all gestational age and weight groups. This, per se, can justify the difference between the results of the present study and the results of Glen’s study.

In another research, all the neonates under study were healthy neonates weighing 2.5 to 3.5 kg and all were full-term, and 78.3% of the neonates were hypothermic, albeit in a mild form [26]; this result shows the importance of temperature management and monitoring even in healthy, full term, and normal weight neonates.

Perez et al. in their study found that the thermoregulation of VLBW (Very Low Birth Weight) neonates is done as routine care for all neonates regardless of other factors such as weight and gestational age [4]. This was an important point that was carefully considered in the present study using the web application for neonatal thermoregulation.

In addition, in most of the studies conducted in the field of thermoregulation of neonates, the focus has been on the cases of hyperthermia or hypothermia. For example, in the field of neonatal hyperthermia, Amadi et al.‘s study showed that 35% of neonates had hyperthermia, and the results of this study also showed that neonatal hyperthermia is related to infant mortality [27]. In another study conducted in 2022, 3.3% of neonates in the neonatal intensive care unit suffered from hyperthermia [28].

The difference in the prevalence and occurrence of hyperthermia can indicate the effect of monitoring, controlling, and appropriate nursing interventions on reducing neonatal temperature instability regardless of any demographic factors such as gestational age, the condition of the neonate, weight, etc. Additionally, in a study conducted to implement a quality improvement project in the neonatal intensive care unit, about 90% of neonates had some degree of hypothermia before the intervention, which significantly decreased after the implementation of the quality improvement program [29]. In the same line, in another study (2017), also conducted on quality improvement, 50% of neonates had hypothermia before the implementation of the project, and after 4 periods of implementing the quality improvement process, 100% of neonates were within the normal temperature range [30]. Thus, it appears that the use of electronic decision support systems similar to the temperature adjustment decision support system used in the present study also can be used in all care fields as a quality improvement project.

## Conclusion

These results indicate that a nurse’s sensitization and guidance with a neonatal thermoregulation decision support system can help to effectively neonate thermoregulation and the nurse has brought the temperature care close to the standard care based on the conditions of each neonate. Also, the results of this study have demonstrated that the use of clinical guidelines, especially in cases where critical care is involved, yields very favorable outcomes in improving the quality of care. On the other hand, electronic and web-based clinical decision support systems can serve as reliable assistants, contributing to both the enhancement of patient care quality and the satisfaction of users(personnel), particularly in clinical settings with severe human resource constraints and limited time for continuous staff training.

### Limitations of the study

In the present study, the quasi-experimental method of conducting the research and the small number of nurses who volunteered to participate in the research were the limitations of the study, which can make it difficult to generalize the results.

### Electronic supplementary material

Below is the link to the electronic supplementary material.

**Supplementary File 1**. Neutral Thermal Environmental Temperatures guideline

## Data Availability

The designed web application can be accessed at the https://thermo.ehms.ir address in any type of browser. The admin’s web application username and password will be sent to you to view different parts of the application. User name: Maryam/Password: 13,441,357. When you enter the web application by clicking on the flag icon, the language of the web application will be changed to English. You can view all parts of the web application based on what is explained in the article. Of course, because this web application was designed and implemented in Iran, the information about the patients is included in the Persian part of the web application. The statistical data was sent to you as part of the research data.
