# Supplementary material for: Development, implementation, and evaluation of neonatal thermoregulation decision support web application
Source: BMC Med Inform Decis Mak. 2023 Oct 18;23:227. doi: 10.1186/s12911-023-02302-4 (PMC10585747; doi:10.1186/s12911-023-02302-4)
Supplement: Supplementary file 1 — Neutral Thermal Environmental Temperatures guideline [file 12911_2023_2302_MOESM1_ESM.docx]

**Supplementary file# 1**

**Supplementary File 1**. Neutral Thermal Environmental Temperatures guideline

| **Age and Weight** | **Range of Temperature (° C)** | **Age and Weight** | **Range of Temperature (° C)** |
| --- | --- | --- | --- |
| **0 to 6 hours** |  | **72 to 96 hours** |  |
| <1200 g | 34 to 35.4 | <1200 g | 34 to 35 |
| 1200 to 1500 g | 33.9 to 34.4 | 1200 to 1500 g | 33 to 34 |
| 1501 to 2500 g | 32.8 to 33.8 | 1501 to 2500 g | 31.1 to 33.2 |
| >2500 g | 32 to 33.8 | >2500 g | 29.8 to 32.8 |
| **6 to 12 hours** |  | **4 to 12 days** |  |
| <1200 g | 34 to 35.4 | <1500 g | 33 to 34 |
| 1200 to 1500 g | 33.5 to 34.4 | 1501 to 2500 g | 31 to 33.2 |
| 1501 to 2500 g | 32.2 to 33.8 | >2500 g |  |
| >2500 g | 31.4 to 33.8 | 4 to 5 days | 29.5 to 32.6 |
| **12 to 24 hours** |  | 5 to 6 days | 29.4 to 32.3 |
| <1200 g | 34 to 35.4 | 6 to 8 days | 29 to 32.2 |
| 1200 to 1500 g | 33.3 to 34.3 | 8 to 10 days | 29 to 31.8 |
| 1501 to 2500 g | 31.8 to 33.8 | 10 to 12 days | 29 to 31.4 |
| >2500 g | 31 to 33.7 | **12 to 14 days** |  |
| **24 to 36 hours** |  | < 500 g | 32.6 to 34 |
| <1200 g | 34 to 35 | 1501 to 2500 g | 31 to 33.2 |
| 1200 to 1500 g | 33.1 to 34.2 | >2500 g | 29 to 30.8 |
| 1501 to 2500 g | 31.6 to 33.6 | **2 to 3 weeks** |  |
| >2500 g | 30.7 to 33.5 | <1500 g | 32.2 to 34 |
| **36 to 48 hours** |  | 1501 to 2500 g | 30.5 to 33 |
| <1200 g | 34 to 35 | **3 to 4 weeks** |  |
| 1200 to 1500 g | 33 to 34.1 | <1500 g | 31.6 to 33.6 |
| 1501 to 2500 g | 31.4 to 33.5 | 1501 to 2500 g | 30 to 32.7 |
| >2500 g | 30.5 to 33.3 | **4 to 5 weeks** |  |
| **48 to 72 hours** |  | <1500 g | 31.2 to 33 |
| <1200 g | 34 to 35 | 1501 to 2500 g | 29.5 to 32.2 |
| 1200 to 1500 g | 33 to 34 | **5 to 6 weeks** |  |
| 1501 to 2500 g | 31.2 to 33.4 | <1500 g | 30.6 to 32.3 |
| >2500 g | 30.1 to 33.2 | 1501 to 2500 g | 29 to 31.8 |
